# On the Design of a Network Digital Twin for the Radio Access Network in 5G and Beyond

**DOI:** 10.3390/s23031197

**Published:** 2023-01-20

**Authors:** Irene Vilà, Oriol Sallent, Jordi Pérez-Romero

**Affiliations:** Signal Theory and Communications Department, Universitat Politècnica de Catalunya (UPC), 08034 Barcelona, Spain

**Keywords:** 5G, Network Digital Twin, Radio Access Network, reinforcement learning, training, network slicing, capacity sharing

## Abstract

A Network Digital Twin (NDT) is a high-fidelity digital mirror of a real network. Given the increasing complexity of 5G and beyond networks, the use of an NDT becomes useful as a platform for testing configurations and algorithms prior to their application in the real network, as well as for predicting the performance of such algorithms under different conditions. While an NDT can be defined for the different subsystems of the network, this paper proposes an NDT architecture focusing on the Radio Access Network (RAN), describing the components to represent and model the operation of the different RAN elements, and to perform emulations. Different application use cases are identified, and among them, the paper puts the focus on the training of Reinforcement Learning (RL) solutions for the RAN. For this use case, the paper introduces a framework aligned with O-RAN specifications and discusses the functionalities needed to integrate the NDT. This use case is illustrated with the description of a RAN NDT implementation used for training an RL-based capacity-sharing solution for network slicing. Presented results demonstrate that the implemented RAN NDT is a suitable platform to successfully train the RL solution, achieving service-level agreement satisfaction values above 85%.

## 1. Introduction

The 5G mobile networks have been designed to provide a wide range of new services and application scenarios (i.e., smart cities, virtual reality, public safety, industry, etc.) with multiple and heterogeneous requirements (i.e., high data rate, low latency, high reliability, etc.) [1]. To achieve this, 5G has incorporated several technological advances covering radio access technologies (e.g., use of millimetre wave bands, the introduction of flexible numerologies, massive Multiple Input Multiple Output (MIMO), etc.) and architectural ones (e.g., network slicing, functional split for base station disaggregation, etc.). The introduction of all these advancements has contributed to an unprecedented level of complexity when it comes to the management of the 5G network. In addition, the vision of future 6G networks as ultra-flexible suggests that the complexity of oncoming networks will be even greater [2]. 

Given this expected high complexity, network operators require tools that allow them to learn all possible outcomes when performing management operations on the network (e.g., network deployment, configuration, optimization, etc.), and thus, find the most appropriate strategies and reduce costs [3]. As a potential solution to address this, the Network Digital Twin (NDT) is identified, which provides a virtual and updated representation of the network that brings the possibility to analyse, diagnose, and emulate the physical network in a zero-risk environment, and based on these, send control decisions to the physical network [4]. Therefore, NDT can allow network operators to explore new techniques and configurations on a safe platform, avoiding the need to perform risky operations on the real network infrastructure. 

The development of NDTs for 5G and beyond 5G (B5G) networks is still in an early stage. Some survey-like papers have provided initial visions on NDTs for B5G [3,4,5,6,7,8,9], identifying the main challenges, potential technologies and use cases of NDTs. Moreover, standardization bodies have also started working on the integration of NDTs. In this regard, IETF has prepared a draft on the NDT concept and architecture in [10], 3GPP opened a studio item on NDT for network management in [11], and ITU has identified the use of NDT for “intent assurance” in [12], where the decisions to be made on the network are validated on an NDT.

The NDT design needs to provide a high-fidelity mirror of the network, to be achieved with an efficient execution time and an efficient use of computational resources, achieving a balance between the NDT complexity and accuracy and providing a high level of flexibility to update the NDT in step with the continuous changes in the physical network [5]. An NDT can involve different subsystems of the network, including the core network and the Radio Access Network (RAN), but also the cloud edge and the transport network that includes fronthaul and backhaul subsystems or even the possibility of having satellite connectivity, as discussed in [2]. At the same time, each subsystem might include different components (e.g., for the case of the backhaul subsystem, node components and fiber links components, etc.). About this, previous works on the use of NDT for 5G networks in [5,6,7,8,9] have only proposed a high-level design of NDTs, mainly distinguishing the main subsystems. Only the work in [3] identified some specific components to be included in an NDT of the whole network. However, the particularities of each of the subsystems of an NDT will require further effort from the research community to specify a proper design.

To the authors’ best knowledge, this paper is the first one to focus on the design of an NDT for representing the RAN subsystem. The applications of NDT for the RAN embrace different RAN management functions, including planning, operation and optimization [5,7]. For planning, NDTs can be used to assess different RAN deployment topologies and configurations before the real deployment is held out, allowing their assessment under different conditions (e.g., traffic load levels). In the case of network operation, NDTs can contribute to real-time monitoring and anomaly detection by using the prediction features of the NDT models. Network optimization processes can benefit from NDTs for tuning different parameter configurations and for exploring new policies to be used by certain optimization functions, such as load balancing, capacity sharing for network slicing, etc. 

Another application of NDTs for the RAN is the training of Machine Learning (ML) solutions [13], which can be used for dealing with multiple problems in different areas of the RAN such as physical layer processing, Medium Access Control (MAC), Radio Resource Management (RRM), Radio Network Management (RNM) or Self-Organizing Networks (SON). Among the ML techniques, Reinforcement Learning (RL) solutions are of special interest in the RAN because they are conceived for optimally solving decision-making problems. As noted by [14], they have been used in the literature for problems such as cell selection, channel selection, resource allocation (scheduling), power allocation, small cell activation/deactivation for energy efficiency, adaptive modulation and coding, etc. 

The use of NDT for training RL algorithms can be the key to the practical adoption of RL solutions in real RANs. This is because RL learning is achieved from the interactions with the environment based on exploitation (i.e., selecting the best actions according to the current learnt policy) and exploration (i.e., selecting some actions randomly to enhance the current learnt policy with the outcomes of new actions). This exploration behaviour becomes one of the key challenges to applying RL algorithms in real RANs, since the fundamentally online trial-and-error learning behaviour may lead to unacceptable degradations in network performance during the learning. In this context, NDT brings the opportunity to train RL algorithms for the RAN on a virtualized, updated and safe version of the real RAN. 

The use of NDTs for training RL solutions for the RAN has been proposed in [15], where the training of a Distributed Deep Q-Network (DDQN) agent that learns the optimal network slicing policy is performed on an NDT. Moreover, the work in [13] proposes a framework for training a RL agent online using an NDT, which is applied for a specific RL-based solution for massive MIMO configuration. While the works in [13] and [15] exemplify the use of NDT for the training of RL solutions, none of the existing works has discussed the capabilities, requirements, procedures and management tasks of an NDT for the training of RL solutions for the RAN. Furthermore, given the standardization efforts of some bodies, such as the O-RAN Alliance, on frameworks for training ML and RL solutions for the RAN, the way to integrate NDTs into these frameworks is a relevant aspect that remains a gap in the literature. This paper covers these two research gaps by proposing the integration of NDTs on current frameworks of O-RAN alliance for training RL solutions and by introducing different functionalities required for the configuration and operation of the NDT to conduct the training.

Therefore, the main novelties and contributions of this paper are twofold: 

First, motivated by the need to further specify the design of an NDT for the RAN subsystem, a novel RAN NDT architecture is proposed. The proposed architecture, which considers the terminology and structure of the NDT proposed by IETF in [10], includes the different components required for building and representing the different RAN entities and the functionalities for gathering real data, managing and operating the NDT. Detailed insights on the component’s requirements and operation of an NDT for the RAN subsystem are provided, as a difference from the prior works in [5,6,7,8,9], which only propose a high-level design of NDTs embracing several subsystems, or the work in [3], which basically identifies the components to be included in a RAN NDT but without providing many details on the component’s requirements. In addition, new functional components that had not been identified in previous works are included in the proposed architecture in this paper regarding mobility, RAN management and optimization, RAN NDT exploitation and NDT management. Moreover, the designed multi-component architecture allows a flexible customization of the RAN NDT to be used in different applications and use cases. 

Second, given the relevance and benefits of using NDT for training RL solutions for the RAN, this NDT use case is addressed in detail in the paper and different functionalities are proposed for supporting the NDT configuration and the training of RL algorithms on it. These functionalities are described in the context of the O-RAN Alliance ML workflow [16]. To the authors’ best knowledge, none of the previous works has proposed the integration of a RAN NDT within the O-RAN framework, nor have the functional requirements been discussed for conducting the training on an NDT. So, this paper presents an illustrative example of the proposed framework for training that consists of the implementation of an NDT for the RAN to conduct the training of a specific RL solution for capacity sharing. The implemented NDT is built following the proposed NDT architecture in the paper. Results on the training of the capacity sharing solution on the implemented RAN NDT are provided, showing the usefulness of the NDT for the RAN as a platform to successfully train RL solutions. 

The rest of the paper is organized as follows. Section 2 presents the architecture of the NDT for the RAN. Then, Section 3 presents a functional model for training RL solutions on an NDT of the RAN. Section 4 presents a specific applicability example of the proposed model illustrated with some results. Finally, conclusions and future work are summarized in Section 5. 

## 2. NDT Architecture for the RAN

**Figure 1:**
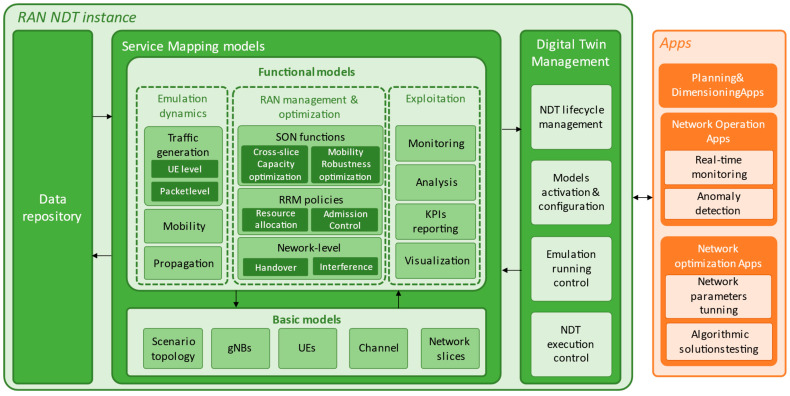
RAN NDT architecture and apps. The RAN NDT architecture includes the data repository, service mapping models to represent the RAN elements and operation, and digital twin management to manage the RAN NDT instance.

The design of the RAN NDT needs to allow the accurate characterization of the real RAN behaviour and its performance assessment, as well as to be flexible for use in different applications and cases. Considering this, Figure 1 shows the architecture of the NDT of the RAN that is proposed in this paper. For facilitating the practical feasibility of the proposed architecture, it is aligned with the terminology and concepts proposed in IETF in [10]. The RAN NDT architecture includes three main modules: the *data repository*, which gathers and stores the data from the real RAN, the *service mapping models*, which allow representing the different elements and the operation in the real RAN, and the *digital twin management*, which manages the NDT. In addition, the architecture identifies different potential applications, or *Apps*, that can benefit from a RAN NDT instance of the proposed architecture customized to its requirements. In the following, further details on these modules are provided.

### 2.1. Data Repository

The *data repository* module is responsible for collecting data from the real RAN environment and storing it to feed the RAN NDT. This allows the NDT to have an accurate and real-time representation of the real RAN but also provides historical data to be exploited by the models in the NDT. 

Types of data included in the repository can be related to configurations, operational states, topology, traces, key performance metrics, etc. The data may also vary in the level of detail (i.e., packet level, time slot level, user level, etc.) as well as in the time scale in which it is gathered (e.g., milliseconds, seconds, minutes, etc.). This will depend on the purpose of the NDT instance (e.g., training a RL model at the MAC layer operating at millisecond time scales or a RRM policy operating in the order of minutes) and the required data by the different models in the NDT. 

### 2.2. Service Mapping Models

The *service mapping models* includes different models for representing the various elements and functionalities in the RAN. A modular design is devised to improve the programmability of the network services and the agility of operation and deployment. The models are fed by the data in the data repository to update their parameters according to the real RAN behaviour. The different service mapping models interact between them and are divided into two main types: basic and functional models, which are described in the following subsections, indicating some considerations on their design.

#### 2.2.1. Basic Models

Basic models refer to RAN network elements and entities that allow capturing the configuration and the RAN environment information. Figure 1 proposes some basic models, namely *scenario topology*, *gNBs*, *UEs*, *channel* and *network slices*. Note that the incorporated models correspond to the required ones to represent a 5G RAN deployment in the NDT, but more models could be incorporated if required (e.g., an eNB model in case of considering interworking with an LTE network).

- *Scenario topology* model: covers the topographic information of the RAN area considered for the NDT, such as detailed maps including buildings, streets, parks etc.
- *gNBs* model: emulates the behaviour of the gNBs (i.e., 5G nodes) including aspects such as their deployment information (e.g., gNB position, gNB height), configuration (e.g., operating frequency, bandwidth configurations, numerologies, beamforming model) and operation (i.e., packet conformance at different levels, etc.).
- *UEs* model: emulates the behaviour of Users Equipment (UEs) capturing physical aspects such as their position, height, antenna gain or the noise figure but also others such as the UE type (e.g., pedestrian, vehicular, sensor) and/or the UE Quality of Service (QoS) requirements (e.g., minimum bit rate, latency, reliability).
- *Channel* model: characterizes the links between the UEs and the gNBs, considering the effect of propagation loss, interference or noise in accordance with the parameters of UEs and gNBs in the NDT (e.g., antenna heights, gains, noise figure, etc.).
- *Network slices* model: for systems with multiple slices, this module embraces all aspects related to their characterization. For each slice, this may include the UEs belonging to the slice, the Service Level Agreement (SLA) to be guaranteed and per-slice parameters to be considered in the scenario.

#### 2.2.2. Functional Models

The functional models cover network analysis, emulation, diagnosis, prediction and assurance. In Figure 1, three subgroups of functional models are proposed.

The first subgroup is the *emulation dynamics*, which refers to those models that allow controlling the RAN environment dynamics in the NDT. The proposed models in this subgroup are the following:

- *Traffic generation* model: allows emulating a realistic behaviour of the traffic demand in the real RAN at different levels (e.g., packet, user, service, network slice or system levels), accounting for the temporal and spatial distributions in the considered RAN area. The traffic generation can be either based on available models in the literature (e.g., session generations according to a Poisson distribution and session durations modelled by an exponential distribution) or on geo-localized traces and measurement reports generated by UEs during their connection to the network. The traffic generation model interacts with the UEs model in Figure 1 to generate new UE sessions.
- *Mobility* model: emulates how the users move in the RAN area, characterizing their trajectories and speed. Different models can be defined for the existing user types in the RAN area (e.g., pedestrian, vehicular, static, drones, etc.). This model interacts with the UEs model to modify the UE’s positions and takes into account the scenario topology model for users’ trajectories (e.g., pedestrian users usually walk on the pavement while vehicles drive on the road).
- *Propagation* model: allows generating different radio propagation conditions, capturing effects such as path loss, attenuations due to diffraction and rain, shadowing or fast fading. The outcomes of the propagation model are provided to the channel model in Figure 1.

The second subgroup is the *RAN management & optimization*. The models belonging to this subgroup are management and optimization models that are applied in the real RAN and, thus, impact the network behaviour. This includes *RRM policies* (e.g., admission control, resource allocation, packet scheduling, etc.), *optimization functions at network level* (e.g., interference coordination, handovers) and *SON functions* (e.g., capacity and coverage optimization, mobility load balancing, cross-slice capacity optimization, etc.). These functions tune and control the different basic models. 

The last group of functional models is the *exploitation,* embracing models for exploiting the outputs of the rest of the *service mapping models*. The proposed models in this group are the *monitoring* model, which allows obtaining detailed information of the functional and basic models in the RAN NDT, the *Key Performance Indicator (KPI) reporting* model, which generates reports with KPI metrics of interest (e.g., resources utilization, throughput, QoS satisfaction, etc.), the *analysis* model, for processing the obtained KPIs to assess the performance of the different NDT models, and the *visualization* model, which can provide different kind of graphs such as the evolution of different KPIs, coverage maps of the different gNBs included in the RAN NDT, etc. 

#### 2.2.3. Models Design

The *service mapping models* can be approached as analytical models, simulators, or ML-based models. The selection of one or another approach can depend on different aspects such as the availability of data (i.e., if there are not any available data, an ML model cannot be trained), the level of complexity of the selected approach or the required accuracy in the NDT. For instance, the traffic generation model in an NDT of the RAN can be approached as a ML model trained based on real data. However, if not enough data are available for the training of the ML approach, the model can also be designed as a simulator, whose parameters can be fine-tuned to adapt to real data collected from the RAN.

### 2.3. NDT Management

The NDT management module in Figure 1 fulfils the management function of the NDT. This includes the NDT’s life-cycle management, i.e., the deployment, operation, optimization, maintenance and termination of the different models in the NDT. This also embraces the feed of data from the data repository to the different models and the validation of the performance of the models as well as the interactions between them. In the case of ML-based models, the NDT management module can provide support along the AI/ML life cycle of the ML-based models in the NDT (i.e., training based on new data, testing and operation). Moreover, the NDT management module is responsible for the activation of the models specified when creating a specific RAN NDT instance. Another functionality is the control of emulation processes on the NDT when it is used for assessing and exploring the network performance in different situations (i.e., what-if situations such as different traffic demand levels, diverse configurations of nodes, etc.). In addition, the NDT management module is in charge of the monitoring of the execution of the NDT modules, encompassing resource consumption, problem detection, connectivity between the RAN NDT and the *Apps* and the experienced traffic.

### 2.4. Apps

The proposed architecture considers that different *Apps* can make use of the RAN NDT. Each *App* is provided with a RAN NDT instance customized according to the specific requirements of the *App*. This customization can embrace the activation of selected *service mapping models*, the specification of the data to gather in the data repository, the definition of the required KPIs, the specification of analysis and visualization capabilities for the *App*, the specification of emulations if required, etc.

Figure 1 identifies different relevant *Apps* related to the different stages of the lifecycle of the RAN that are identified as main use cases for the RAN NDT:

- *Planning & Dimensioning Apps*: When deploying new gNBs in the RAN or configuring the existing ones, emulations can be performed on a RAN NDT instance to determine the most adequate gNBs deployment locations and configurations to enhance the coverage and capacity of the network. For this kind of *App*, the NDT instance will include the already deployed gNBs and their configurations. Then, diverse emulations associated with different configurations of the new gNB can be conducted on the RAN NDT instance.
- *Network Operation Apps*: During the operation of the network, a RAN NDT instance can contribute to the monitoring of the network by extending the performance metrics obtained from the real network with additional ones obtained from emulations on the RAN NDT. Based on this, possible failures or anomalies can be predicted and actions can be taken to avoid them. Therefore, the RAN NDT instance for this *App* should include all the elements (scenario topology, gNBs, UE characterization, etc.) as in the real RAN, which will be fed with real-time data from the RAN.
- *Network Optimization Apps:* The optimization of the RAN can benefit from a RAN NDT instance for emulating different RAN configurations as well as for testing algorithmic solutions before they are applied in the real RAN, allowing adjustment and validation of their parameters. The testing of configurations can be conducted by enforcing them on the RAN NDT instance and performing emulations. In the case of algorithmic solutions, whether they are based on heuristics, analytics or machine learning, they should be integrated within the RAN NDT instance to assess their performance through emulations. For instance, if a RRM policy for admission control is tested, it should be loaded as a functional model for *RAN management & optimization*, and the corresponding admission decisions should be made on the RAN NDT instance models to assess its performance. Furthermore, note that in the case of ML-based solutions, and specially RL-based solutions, their training can be performed on the NDT instance, as discussed in the next section.

## 3. NDT for RL Training Process

NDT allows performing the training of RL solutions for the RAN. The training process of a RL model consists of learning the optimal policy that selects the best decision (i.e., action) for each possible situation (i.e., state), which is the one that obtains the maximum reward. To achieve this, during the training process a RL agent (i.e., the learner) iteratively interacts with an environment, where at each iteration the RL agent obtains a state, selects an action, and then, as a result of the last action, receives a reward that evaluates how good or bad the last action was for the last state, along with the new state [17]. Actions are selected according to both exploitation, where actions are selected following the RL agent’s policy, and exploration, where actions are selected randomly. From this interaction, the RL agent updates the policy. This is repeated until a convergence condition is reached, when the training process is terminated. 

RL solutions can be applied to problems of the RAN involving some sort of decision making, mainly associated with RRM, RNM or SON functions. RL solutions for RRM functions dynamically manage the provisioned resources operating at different time scales from milliseconds to a few seconds and cover solutions for the different layers of the protocol stack. Examples of RL applicability for RRM functions are channel coding, power control and dynamic spectrum access at the physical layer, scheduling at the MAC layer, admission control for Radio Resource Control (RRC) and dual connectivity at the Packet Data Convergence Protocol (PDCP) layer. Moreover, RL can be applied to RNM and SON functions to support the network planning, deployment and operation of the RAN in terms of configuration, optimization and fault management, operating at longer-term time scales. For instance, RL applicability examples related to SON functions include capacity and coverage optimization, mobility robustness optimization, mobility load balancing or cross-slice capacity optimization, also referred to as capacity sharing, for assigning available resources to the different slices.

Despite the wide applicability of RL for the RAN, the training of these solutions represents a relevant challenge from the implementation perspective. This is because the trial-and-error behaviour involved during the training of RL solutions can lead to unacceptable degradations in network performance if the training is performed directly on the real RAN. To address this problem, the training of RL can be safely performed on a RAN NDT. This can be conducted by considering the RL solution as an *App* in Figure 1 that interacts with a RAN NDT instance during the training.

**Figure 2:**
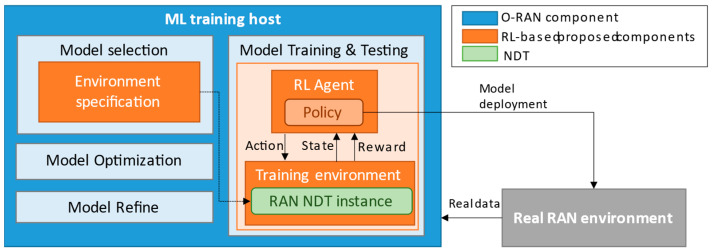
Functional model of the RL model training process on NDT. The training is performed on the ML training host that includes different functionalities identified in the O-RAN ML workflow.

Figure 2 includes the proposed functional model for the training of RL models for the RAN by using a RAN NDT. To facilitate its practical feasibility, the model is aligned with the main components and functionalities involved in the training of ML models in the O-RAN Alliance ML workflow [16]. These functionalities are coloured blue in Figure 2. As proposed components in this paper, the functional model includes a RAN NDT instance, which is coloured green, and different components to support the training of the RL-based models on it, coloured orange. 

The training of RL models is performed at the ML training host, which includes different components, namely, *model training & testing, model selection, model optimization* and *model refine*, all of them proposed in the O-RAN Alliance ML workflow [16]. 

Among these ML training host components, the RL model training is carried out by the RL agent under the *model training & testing*, where the RL policy iteration process takes place through the iterative interaction of the RL agent with the RAN NDT instance that constitutes the training environment, making use of exploration and exploitation. Hence, along the training process the RL agent iteratively obtains the states and rewards from the RAN NDT instance and sends the actions to it. Then, according to the states, actions and rewards, the policy is updated. The specific algorithm for updating the policy depends on the considered RL technique (e.g., for Q-learning and DQN techniques the corresponding algorithms can be found in [17,18], respectively). Note that the states and rewards will result from the sent action, but also from the emulation dynamics and components’ configurations in the RAN NDT instance. During the training, different KPIs are monitored to assess the model convergence and determine whether the training process is completed. Afterwards, the model testing takes place, consisting of validating the behaviour of the trained model. This is performed by selecting the actions according to the trained policies under exploitation mode and by triggering them on the RAN NDT instance. During this process, the performance of the trained policies can be assessed through different RAN-related KPIs (e.g., throughput, error rates, etc.). Once the training and validation are completed, the RL model (i.e., the trained policy) can be packaged up, e.g., as a container, and be deployed on the real RAN environment, where the RL model actions are executed during a stage known as *inference*. 

Within the ML training host, the *model selection* function selects the configurations for the inference and the training of the RL model based on the requirements of the RL solution e.g., training accuracy, training time, hardware resources in training and inference, inference speed, etc. To control and configure the training environment, the *environment specification* function is proposed to be included in the *model selection* functionality. Specifically, this function configures the RAN NDT instance by specifying the models of the RAN NDT instance that are required for the training of the RL model. To this end, the *environment specification* interacts with the *NDT management* module in Figure 1 to configure the required models in the *model activation & configuration* functionality. For example, this might include the activation and configuration of the gNB and scenario topology models in Figure 1 of the RAN NDT instance according to the gNBs configurations and topology in the real RAN, respectively. Moreover, to ensure high performance of the RL model training, the RL agent needs to experience, during the training, those relevant situations (i.e., states) that will be experienced later on during inference. The *environment specification* function is also responsible for the configuration of the RAN NDT instance parameters to emulate these situations in the *emulating running control* functionality of the *NDT management*. For instance, different traffic levels or load distributions among the different gNBs can be set up for emulation. As a RAN NDT instance can be fed by data from the real RAN environment to be used by the *service mapping models* shown in Figure 1, another responsibility of the *environment specification* function is to specify the data needed by the RAN NDT instance. Based on this configuration, data are gathered from the real RAN environment and stored in the *data repository* of the RAN NDT instance. 

Regarding the *model optimization* component in the ML training host, it allows the optimization of the hyperparameters of the RL model (e.g., number of layers of a neural network in case of deep RL, number of neurons per layer, etc.) based on certain hardware or performance metrics requirements such as model accuracy, model size, inference speed, memory used, etc. Then, the RAN NDT instance can be used to test the performance of different policies associated with different hyperparameter configurations. 

Finally, the *model refine* component allows, if required, the upgrade of the model through re-training after it has already been deployed in the real RAN. This can be required when the performance of the deployed policies is no longer optimal due to mismatches between the conditions considered during training and those in the real RAN environment. For instance, this can occur when changes are made to the gNBs model (e.g., new gNB deployment) or its elements configuration (e.g., a change in the configuration of a gNB), or also when the users in the real RAN start behaving differently (e.g., new UE requirements, new services are offered in the real RAN, etc.). In these situations, the *model refine* component is notified and, accordingly, it triggers a re-training process conducted on a RAN NDT instance updated in accordance with the new conditions. 

## 4. Results

In this section, a use case example of the training of RL solutions on a RAN NDT is provided. The considered use case is a RL solution for capacity sharing in RAN slicing, as described in Section 4.1. Then, an implementation of a RAN NDT for the training of the RL-based capacity sharing solution is described in Section 4.2 that is based on the RAN NDT architecture proposed in Section 2. Next, the performance evaluation is assessed in Section 4.3 that focuses on the training of the RL-based capacity sharing solution on the implemented RAN NDT and on the performance of the learnt policies after training. 

### 4.1. Use Case Example 

This section presents an implementation of a RAN NDT to train an RL-based capacity sharing solution for RAN slicing based on the architecture proposed in Figure 1. 

The RL-based capacity sharing solution has the role of an *App*, as shown in Figure 1. Specifically, the considered capacity sharing solution is the Deep Q-Network Multi-Agent-Reinforcement Learning (DQN-MARL) capacity sharing approach proposed in our work in [19]. The solution allows dynamically distributing the available capacity in a RAN infrastructure composed of *N* gNBs among *K* tenants, each of them provided with a RAN slice. Each gNB *n* has a total capacity of *c_n_* (b/s). The solution targets the efficient use of the available capacity in the gNBs and, at the same time, the satisfaction of the SLA of the tenants. The SLA established for the *k*-th tenant is defined in terms of: (a) the Scenario Aggregated Guaranteed Bit Rate, *SAGBR_k_*, which is the aggregated capacity to be provided across all gNBs to tenant *k* if requested, and (b) the Maximum Cell Bit Rate, *MCBR_k,n_*, which is the maximum bit rate that can be provided to tenant *k* in cell *n*. The DQN-MARL capacity sharing solution considers that each tenant is associated to a different RL agent. Each agent tunes the resource quota (i.e., the fraction of capacity) assigned to the tenant’s slice in the different gNBs in time steps of duration Δ*t*, in the time scales in the order of minutes. 

To learn the policy that tunes the resource quota for each tenant, at each time step, the RL agent obtains the state of the tenant in the different gNBs of the environment, which is defined as a tuple with different metrics, including the resource usage and resource quota of the tenant, the resource quota not assigned to any tenant, the resources not used in the gNB and the SLA parameters of the tenant. According to the obtained state, the RL agent decides the actions to perform in each gNB, which can be to increase the resource quota in Δ, to decrease it in Δ or to keep it unaltered. To assess to what extent the applied resource quota in each gNB was suitable, a reward is provided to each RL agent in the following time step. The reward definition promotes the satisfaction of the SLA parameters and the minimization of overprovisioning situations. For further details on definitions of the state action and reward, the reader is referred to [19]. During the training of the solution, the RL agents interact with a RAN NDT instance, from which the state and reward signals are obtained and the actions, selected by an ε-Greedy strategy, are applied in the different gNBs. Based on this interaction, the policy that tunes the resource quotas is updated until it reaches convergence.

### 4.2. RAN NDT Instance

The implemented RAN NDT for training the DQN-MARL capacity sharing solution corresponds to a specific RAN NDT instance of Figure 1. Following the functional model in Figure 2, the *environment specification* module in the training host specifies the *service mapping models* of the RAN NDT instance and their configuration to emulate different realistic RAN conditions (i.e., RL agent states), apply the RL actions and obtain the rewards. In particular, the implemented RAN NDT instance allows capturing realistically the spatial traffic distributions in an area with different gNBs and emulates the network behaviour under different dynamic conditions with UEs moving through the scenario and generating traffic. In the following, the description of the basic and functional models in the implemented NDT (i.e., RAN NDT instance) is provided. 

#### 4.2.1. Basic Models

**Figure 3:**
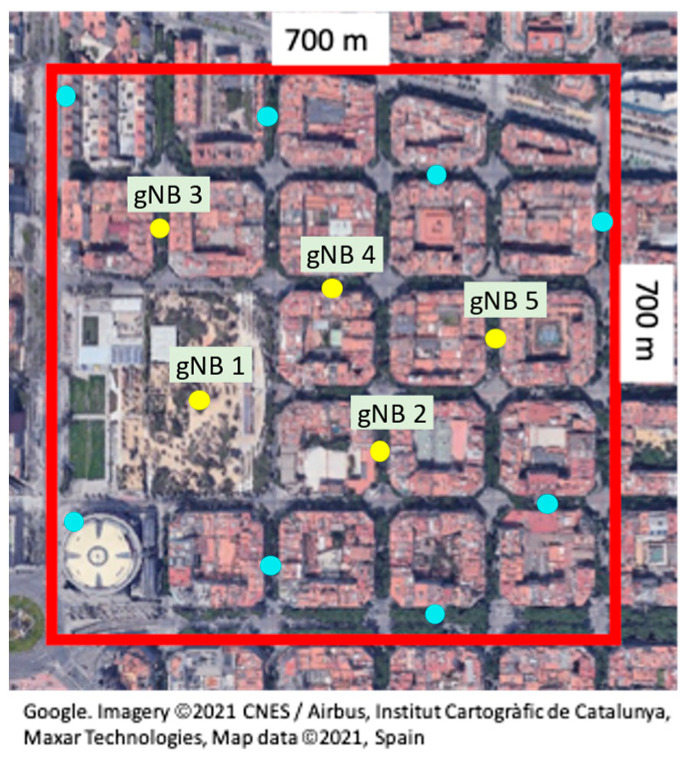
Map included in the scenario topology model of the implemented RAN NDT. The positions of the considered gNBs correspond to the yellow dots on the map.

The implemented NDT has approached the basic models as follows:

1. *Scenario topology* model: corresponds with an urban scenario of 700 × 700 m in Barcelona city shown in Figure 3, which encompasses different streets, avenues, a park, and different seven-floor buildings with 3.5 m floor height.
2. *gNB* model: includes the gNBs deployed in the area. Each one is modelled as an outdoor microcell and is specified by the following parameters: gNB position, height, operating frequency, total transmitted power, total bandwidth, subcarrier separation, total number of Physical Resource Blocks (PRBs), duplex mode and the gNB antenna gain and noise figure. Only the downlink (DL) direction is considered. The positions of the gNBs in the NDT are shown in Figure 3. To avoid border effects, it is assumed that the study of the capacity sharing algorithm is performed only in the five central gNBs that are numbered and highlighted in yellow in the figure.
3. *UE* model: is specified by the user density in the area (users/km^2^), the UE type, which can be either stationary, pedestrian or vehicular, as well as by the UE position that changes dynamically, the UE height, antenna gain, noise figure and required bit rate *R_b_*.
4. *Channel* model: provides the Signal-to-Interference and Noise (SINR) ratio for each UE by considering the UE and gNB parameters and obtaining the path loss according to the UE position and the propagation model of the NDT. Moreover, the channel model provides the spectral efficiency *S_eff_* in b/s/Hz to each UE according to the SINR and assuming that the maximum achievable *S_eff_* is 7.4063 b/s/Hz as established in [20], and that UE is in outage if *S_eff_* is lower than 1 b/s/Hz. Additionally, inefficiency factors due to cyclic prefix of 5G transmission and overheads (see [22]) have been considered, taking values 14/15 and 0.8, respectively.
5. *Network slice* model: is specified by the associated SLA to be guaranteed to the different network slices (i.e., the *SAGBR_k_* and *MCBR_k_* parameters in the DQN-MARL capacity sharing algorithm), the bit rate requirement of UEs belonging to the network slice *R_b_*, the traffic generation parameters for the network slice and the percentage of users of the different types in the network slice. In addition, the resource quota of the network slice in the different gNBs during the current time step is also configured here.

The specific values of the parameters considered for the different basic models in the NDT are listed in Table 1.

#### 4.2.2. Functional Models

Concerning the functional models that have been implemented in the NDT, the following have been considered:

(1) *Traffic generation* model: considers that sessions are generated following a Poisson distribution with a rate *λ* (sessions/s) and session durations are modelled by an exponential distribution with average *μ* (seconds). The traffic generation parameters (*λ, μ*) are provided by the network slice model so that the traffic generation is performed per network slice.
(2) *Mobility* model: it is applied for each UE in the RAN NDT and its outcomes modify the position feature in the UE. It is implemented as a random walk model with a certain probability of changing direction in a range (+α, −α) (degrees). The implementation considers that pedestrian UEs move at 3 km/h along the sidewalks of the streets with a 20% probability of changing direction at an intersection. When they are around a park area, they follow a random walk model in which the UE maintains the same direction for an exponentially distributed time with an average of 10 s and makes random direction changes in a range (+45°, −45°) with respect to the current direction. Stationary UEs remain static throughout a simulation and can be placed either outdoors on a pavement or a park or indoors on any of the floors of a building. Vehicular UEs move along the streets at 30 km/h with a 25% probability of changing direction at an intersection.
(3) *Propagation* model: the one included generates a propagation loss map by following the Urban Microcell (UMi) model of [21], which specifies the path loss and 2D correlated shadowing for outdoor-to-outdoor and outdoor-to-indoor links.
(4) *RAN management & optimization* models: the included models of this type in the RAN NDT are the following. First, a *capacity sharing SON* model is included that receives the resource quotas from the RL agents in the DQN-MARL capacity sharing solution and configures them in each gNB. Second, a *resource allocation* model is included as a RRM policy that computes the allocated PRBs, which correspond to the radio resources in 5G, in each gNB to the different UEs according to the resource quotas per network slice provided by the capacity sharing SON function. This resource allocation is computed in time scales of the order of seconds, so effects related to ms time scales (e.g., fast fading, scheduling, etc.) are characterized in average terms. Third, a *cell selection* model is also incorporated as an RRM policy that selects the gNB to be attached by a UE in every NDT execution step. In particular, it is assumed that a UE attaches to the gNB with the maximum SINR.

Moreover, the models to exploit the outcomes from the rest of the functional and basic models in the implemented RAN NDT are the following ones:

(1) *Analysis* model: computes the state and reward components for the RL agents in the DQN-MARL capacity sharing solution.
(2) *KPI metrics* model: provides the generated offered loads, the radio resources utilization, throughput, QoS/SLA satisfaction provided at the system, gNB and slice levels.
(3) *Visualization* model: allows visualizing coverage and traffic density maps in the area as well as plots of the evolution of the different KPIs.

### 4.3. Performance Evaluation

The assessment of the performance of the training of the DQN-MARL capacity sharing solution on the implemented RAN NDT is provided in this section. To conduct the training, the *environment specification* functionality in Figure 2 has provided the model configuration values in Table 1 to the *models activation & configuration* functionality of the implemented RAN NDT. The scenario consists of two tenants, denoted as Tenant 1 and Tenant 2, that share the capacity of 5 gNBs. Moreover, the *environment specification* functionality sets the emulation parameters in the *emulating running control* functionality of the implemented RAN NDT according to the values included Table 2 in terms of the user density in the RAN area for the two considered tenants, the percentage of users of each type (i.e., stationary, pedestrian or vehicle) at morning, afternoon and night and the traffic generation parameters. The parameters considered for Tenant 1 correspond to a business profile, which has more density of users during the morning, while those considered for Tenant 2 correspond to a residential profile, which is more intense during the afternoon. The RAN NDT execution for the training is performed by repeatedly generating different random realizations of traffic generation and mobility following the parameters in Table 2. The emulation in the RAN is performed in time steps of 1 s duration, while the monitoring of the different models is performed by averaging the different traffic-related metrics every 3 min.

**Figure 4:**
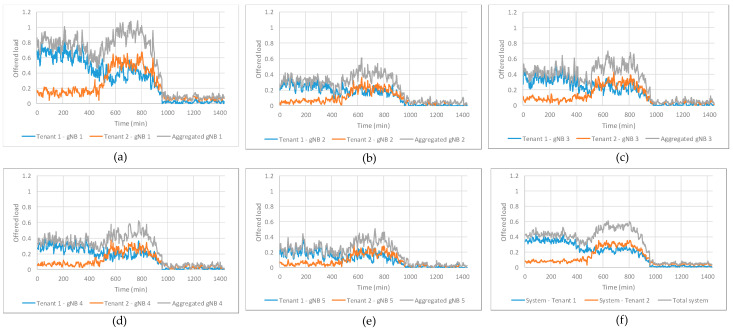
Offered loads per tenant and aggregated for (**a**) gNB 1, (**b**) gNB 2, (**c**) gNB 3, (**d**) gNB 4, (**e**) gNB 5 and (**f**) at system level.

The considered configuration for the DQN-MARL capacity sharing solution is given by the parameters of Table 3. For details on the meaning of each parameter, the reader is referred to [19]. Based on this, the training is performed through the interaction of the RL agents in the DQN-MARL capacity sharing solution with the RAN NDT according to an ɛ-Greedy policy. Moreover, the RAN NDT is configured to provide an evaluation of the trained policy every 5000 steps of training, where a step is one interaction of the RL agents with the RAN NDT instance. This evaluation consists in the application of the trained policy until that moment without considering random actions (i.e., a greedy policy) to a specific realization of the morning–afternoon–night pattern of Table 2 that is shown in Figure 4. The subplots from (a)-(e) depict the evolution of the offered load (i.e., required bit rate) per tenant and the aggregated offered load of both tenants in gNBs 1 to 5, respectively, both normalized to the average gNB capacity. In all the gNBs and according to the emulation configuration in Table 2, the offered load of Tenant 1 is larger during the morning (*t* = 0 min until *t* = 480 min in Figure 4), while it is reduced approximately by 40% during the afternoon (*t* = 480 min until *t* = 960 min in Figure 4) and it is very low during the night (*t* = 960 min until *t* = 1440 min in Figure 4). Instead, the offered load of Tenant 2 remains low during the morning and night, but experiences similar offered load levels to Tenant 1 during the afternoon. Focusing on the aggregated offered loads in each gNB, gNB 1 experiences overload sometimes during the afternoon (i.e., the aggregated offered load is larger than 1), while gNBs 2–5 always have enough capacity to fulfil the offered loads of both tenants. This is because gNB 1 is located in a park (see Figure 3) with higher affluence than the rest of the gNBs. Moreover, subplot (f) in Figure 4 shows the aggregated offered load in the system (i.e., among all the gNBs) per tenant, jointly with the aggregated offered load of both tenants in the system, both normalized to the total system capacity. Note that the aggregated offered loads in the system are generally below the *SAGBR_k_* of both tenants (i.e., 50% of system capacity for Tenant 1 and 30% of system capacity for Tenant 2), only exceeding it sporadically in the case of Tenant 2 during the afternoon.

**Figure 5:**
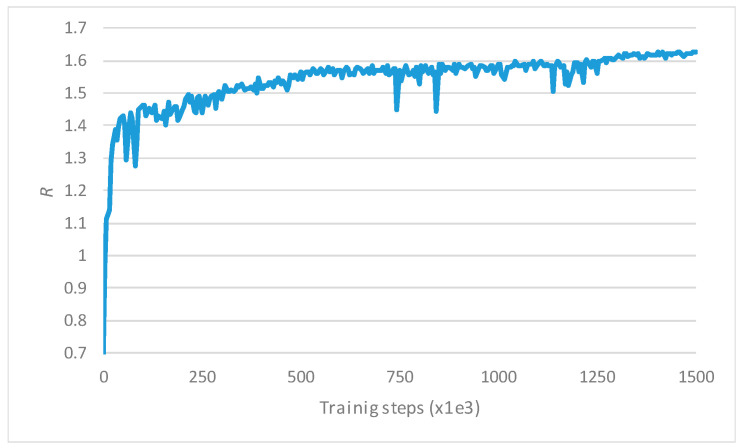
Evolution of the aggregated reward by tenant 1 and tenant 2 during training.

As an output of the *visualization* model in the RAN NDT, Figure 5 depicts the evolution of the aggregated average reward of both tenants, denoted as *R*, as a result of the evaluations conducted periodically during the training. The figure shows that during the first 250·10^3^ training steps, *R* increases abruptly, experiencing large fluctuations. After this period, *R* increases in a slower slope with lower fluctuations until it stabilizes at a value of around 1.6 after approximately 1500·10^3^ training steps. These results highlight the usefulness of the implemented RAN NDT to conduct the training of the DQN-MARL capacity sharing solution.

**Figure 6:**
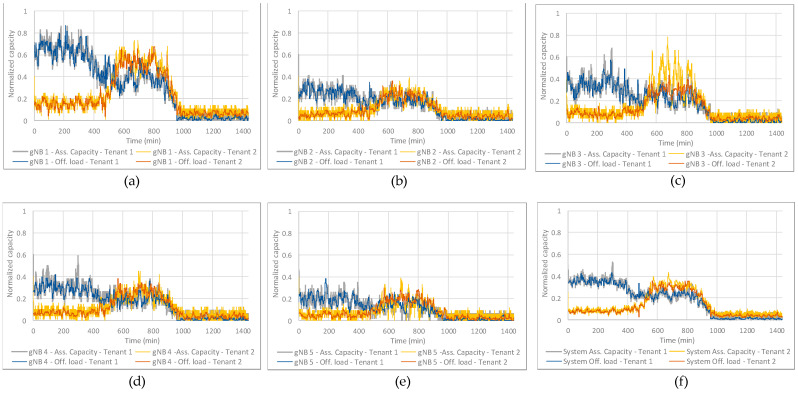
Comparison of the offered loads and the assigned capacity per tenant for (**a**) gNB 1, (**b**) gNB 2, (**c**) gNB 3, (**d**) gNB 4, (**e**) gNB 5 and (**f**) at system level. The required capacities are generally satisfied, showing that the policies have been successfully trained on the RAN NDT.

Moreover, the *visualization* model in the RAN NDT also provides Figure 6, which compares the assigned capacity per tenant by the learnt policy after convergence to their respective offered loads in the different gNBs (subplots (a)–(e)) and system level (subplot (f)). In subplots (a)–(e) both the offered loads and assigned capacities are normalized to the gNB capacity. The results in these subplots illustrate that the assigned capacity per cell adapts to the offered load in all gNBs. Moreover, subplot (f) compares the offered loads and assigned capacities to both tenants in aggregated terms over all gNBs, both normalized to the system capacity. From this subplot, the fact that the learnt policies allow satisfying the *SAGBR_k_* requirement can be observed as the aggregated offered loads per tenant, which are lower than their respective *SAGBR_k_*, are satisfied. Hence, the learnt policies on the implemented RAN NDT allow adapting the assigned capacity to the offered loads while satisfying the SLA of both tenants.

To further assess the performance of the learnt policies, the *KPI metrics* model of the implemented RAN NDT provides the per-tenant KPIs in Table 4 in terms of the average reward, the average SLA satisfaction (i.e., ratio between the tenant’s throughput and the minimum between the aggregated offered load in all gNBs and the *SAGBR_k_*) and the average assigned capacity utilization per gNB (i.e., ratio between the throughput and the assigned capacity per tenant). The values in Table 4 show that a similar average reward is obtained by both tenants. In addition, high average SLA satisfaction is achieved, with values above 0.85 for both tenants. The average assigned capacity utilization in all the gNBs is around 0.8, revealing that the learnt policies assign the capacities to both tenants with low overprovisioning. Overall, the obtained KPI values reflect that the implemented RAN NDT has allowed the learning of high-performance policies. 

## 5. Conclusions 

This paper has discussed on the design and applicability of a Network Digital Twin (NDT) for the Radio Access Network (RAN) subsystem. Aligned with the terminology on NDT introduced by IETF, the paper has proposed the architecture of a RAN NDT that is composed of different components for the gathering of data from the real RAN, for the representation of the different entities and functionalities of the RAN, for exporting relevant metrics and for the management of the RAN NDT. Based on the proposed RAN NDT architecture, the specific RAN NDT use case of training Reinforcement Learning (RL) algorithms for the RAN is described and different functionalities aligned with the O-RAN Alliance ML workflow are proposed to support the training on the RAN NDT. 

Moreover, the paper has described an illustrative example of an implemented RAN NDT for the training of a specific RL solution for capacity sharing, namely, the DQN-MARL capacity sharing solution, which allows the dynamic distribution of capacity in scenarios with multiple gNBs among multiple tenants. Results to illustrate the performance evaluation of the training of the solution have been provided for a specific configuration of the RAN NDT. Results show that the training of the solution is successfully performed, which proves the suitability of the implemented RAN NDT as a platform to conduct the training. Also, the learnt policies after training achieve high performance, satisfying the traffic demands of both tenants with low levels of overprovisioning and high service-level agreement satisfaction.

## Figures and Tables

**Table 1:** Configuration of the implemented RAN NDT.

Parameter		Value
*gNB*		
Number of gNBs		5
gNB height		10 m
Operating frequency		3.5 GHz
Total transmitted power		35 dBm
Total bandwidth		100 MHz
Subcarrier separation		30 kHz
Duplex mode		Time Division Duplex (75% of symbols allocated to DL)
Number of PRBs per gNB		273
Antenna gain		20 dB
Noise Figure		9 dB
*UE*		
UE height		1.5 m
UE antenna gain		0 dB
Noise Figure		9 dB
*Channel*		
Average Spectral efficiency (bits/s/Hz)	gNB 1	3.71
	gNB 2	3.73
	gNB 3	3.59
	gNB 4	3.66
	gNB 5	3.59
*Network slice*		
Number of tenants		2
Required user bit rate (*R_b_*)	Tenant 1	8 Mb/s
	Tenant 2	4 Mb/s
*SAGBR_k_*	Tenant 1	50% of the capacity in the system
	Tenant 2	25% of the capacity in the system
*MCBR_k_*	Tenant 1 and 2	90% of the cell capacity

**Table 2:** Emulation parameters for training.

Parameter		Morning (8–16 h)	Afternoon (16–24 h)	Night (0–8 h)
User density (users/km^2^)	Tenant 1	3000	2000	100
	Tenant 2	1000	4000	500
User types percentages	Stationary	50%	60%	90%
	Pedestrian	40%	30%	5%
	Vehicle	10%	10%	5%
Traffic generation parameters	Session generation rate (*λ)*	1 session/s		
	Average session duration (*μ*)	300 s		

**Table 3:** Configuration of the hyperparameters of the DQN-MARL capacity sharing solution.

Parameter	
Initial collect steps	80,000
Maximum number of time steps for training	1.5·10^6^
Experience Replay Buffer length (*l*)	10^7^
Mini-batch size (*J*)	256
Learning rate (τ)	0.0001
Discount factor(γ)	0.9
ɛ value (ɛ-Greedy)	0.1
DNN configuration	Input layer: 17 nodes 1 full connected layer: 100 nodes Output layer: 243 nodes
Reward weights (*φ_1,_ φ_2_)*	(0.5. 0.4)
Action step (Δ)	0.03
Time step (Δ*t*)	30 s

**Table 4:** KPI metrics obtained from *KPI metrics model*.

Parameter		Tenant 1	Tenant 2
Average reward		0.78	0.84
Average SLA satisfaction		0.88	0.90
Assigned capacity utilization	gNB 1	0.83	0.88
	gNB 2	0.77	0.82
	gNB 3	0.78	0.80
	gNB 4	0.77	0.83
	gNB 5	0.78	0.80

## Data Availability

Not applicable.
